# Supplementary material for: Repeated sampling of different individuals within the same clusters to improve precision of longitudinal estimators: the DISC design
Source: Int J Epidemiol. 2026 Jul 9;55(4):dyag108. doi: 10.1093/ije/dyag108 (PMC13348717; doi:10.1093/ije/dyag108)
Supplement: dyag108_Supplementary_Data [file dyag108_supplementary_data.pdf]

# Supplementary Materials

**Repeated sampling of different individuals within the same clusters to improve precision of longitudinal estimators: the DISC design**

## 1 Variance calculations

The estimator given in (2) is defined as

$$\begin{aligned}\hat{\delta} \equiv & \left\{\frac{2}{n}\sum_{i\in I_2}\sum_{k\in K_{i2}} X_i Y_{i2k} - \frac{2}{n}\sum_{i\in I_1}\sum_{k\in K_{i1}} X_i Y_{i1k}\right\} \\ & - \left\{\frac{2}{n}\sum_{i\in I_2}\sum_{k\in K_{i2}} (1-X_i) Y_{i2k} - \frac{2}{n}\sum_{i\in I_1}\sum_{k\in K_{i1}} (1-X_i) Y_{i1k}\right\}.\end{aligned}$$

Assume that the population of clusters is sufficiently large such that the probability of selecting the same cluster at both time points is zero (a simplifying assumption to ease calculations). Then under the RCS design, labelling our estimator as $\hat{\delta}_{\text{RCS}}$, it holds that

$$\begin{aligned}\text{Var}\left(\hat{\delta}_{\text{RCS}}\right) = & \text{Var}\left[\left\{\frac{2}{n}\sum_{i\in I_2}\sum_{k\in K_{i2}} X_i Y_{i2k} - \frac{2}{n}\sum_{i\in I_1}\sum_{k\in K_{i1}} X_i Y_{i1k}\right\}\right. \\ & \left. - \left\{\frac{2}{n}\sum_{i\in I_2}\sum_{k\in K_{i2}} (1-X_i) Y_{i2k} - \frac{2}{n}\sum_{i\in I_1}\sum_{k\in K_{i1}} (1-X_i) Y_{i1k}\right\}\right] \\ = & 2\,\text{Var}\left\{\frac{2}{n}\sum_{i\in I_2}\sum_{k\in K_{i2}} X_i Y_{i2k} - \frac{2}{n}\sum_{i\in I_2}\sum_{k\in K_{i2}} (1-X_i) Y_{i2k}\right\} \\ = & 2\,\text{Var}\left\{\frac{2}{n}\sum_{i\in I_2}\sum_{k\in K_{i2}} (2X_i - 1) Y_{i2k}\right\} \\ = & \frac{8}{n^2}\left[\sum_{i\in I_2}\left\{(2X_i-1)^2 \text{Var} \sum_{k\in K_{i2}} Y_{i2k}\right\}\right] \\ = & \frac{8}{n^2}\left[\sum_{i\in I_2}\left\{\text{Var} \sum_{k\in K_{i2}} (\mu_2 + \delta X_{ij} + \alpha_i + \epsilon_{ijk})\right\}\right] \\ = & \frac{8}{n^2}\left[\sum_{i\in I_2}\left\{\text{Var}(m\alpha_i) + \sum_{k\in K_{i2}} \text{Var}(\epsilon_{ijk})\right\}\right]\end{aligned}$$

$$
\begin{aligned}
&= \frac{8m}{n^2}\left[\sum_{i\in I_2}\left\{m\tau^2+\sigma^2\right\}\right] \\
&= \frac{8\left(m\tau^2+\sigma^2\right)}{n}\,.
\end{aligned}
$$

Under the DISC design, we can label this estimator as $\hat{\delta}_{\text{DISC}}$, and making a similar simplifying assumption that the probability of sampling the same individual at both time points is zero, it holds that

$$
\begin{aligned}
\text{Var}\left(\hat{\delta}_{\text{DISC}}\right) &= \text{Var}\Bigg[\left\{\frac{2}{n}\sum_{i\in I_2}\sum_{k\in K_{i2}} X_iY_{i2k} - \frac{2}{n}\sum_{i\in I_1}\sum_{k\in K_{i1}} X_iY_{i1k}\right\} \\
&\qquad - \left\{\frac{2}{n}\sum_{i\in I_2}\sum_{k\in K_{i2}}(1-X_i)Y_{i2k} - \frac{2}{n}\sum_{i\in I_1}\sum_{k\in K_{i1}}(1-X_i)Y_{i1k}\right\}\Bigg] \\
&= \frac{4}{n^2}\text{Var}\Bigg[\sum_{i\in I_1} X_i\left(\sum_{k\in K_{i2}} Y_{i2k} - \sum_{k\in K_{i1}} Y_{i1k}\right) \\
&\qquad - \sum_{i\in I_1}(1-X_i)\left(\sum_{k\in K_{i2}} Y_{i2k} - \sum_{k\in K_{i1}} Y_{i1k}\right)\Bigg] \\
&= \frac{4}{n^2}\text{Var}\Bigg[\sum_{i\in I_1} X_i\left(\sum_{k\in K_{i2}} (\delta+\epsilon_{i2k}) - \sum_{k\in K_{i1}} \epsilon_{i1k}\right) \\
&\qquad - \sum_{i\in I_1}(1-X_i)\left(\sum_{k\in K_{i2}} \epsilon_{i2k} - \sum_{k\in K_{i1}} \epsilon_{i1k}\right)\Bigg] \\
&= \frac{4}{n^2}\text{Var}\sum_{i\in I_1}(2X_i-1)\left(\sum_{k\in K_{i2}} \epsilon_{i2k} - \sum_{k\in K_{i1}} \epsilon_{i1k}\right) \\
&= \frac{4}{n^2}\sum_{i\in I_1}\left(\sum_{k\in K_{i2}}\sigma^2 + \sum_{k\in K_{i1}}\sigma^2\right) \\
&= \frac{8\sigma^2}{n}\,.
\end{aligned}
$$

## 2 Additional simulation results

In this section, we display additional simulation results that examine sensitivity of results to changes in the data-generating mechanism. Figure S1 shows complete simulation results comparing the empirical variance of the linear estimator to the theoretical variance under the DISC design versus

the RCS design. Results are shown for four cluster sizes (10, 25, 50, and 100) and four ICC values (0.01, 0.05, 0.1, 0.2). These results confirm that the simulation-based empirical variance matches the analytical variance across a variety of data-generating mechanisms.

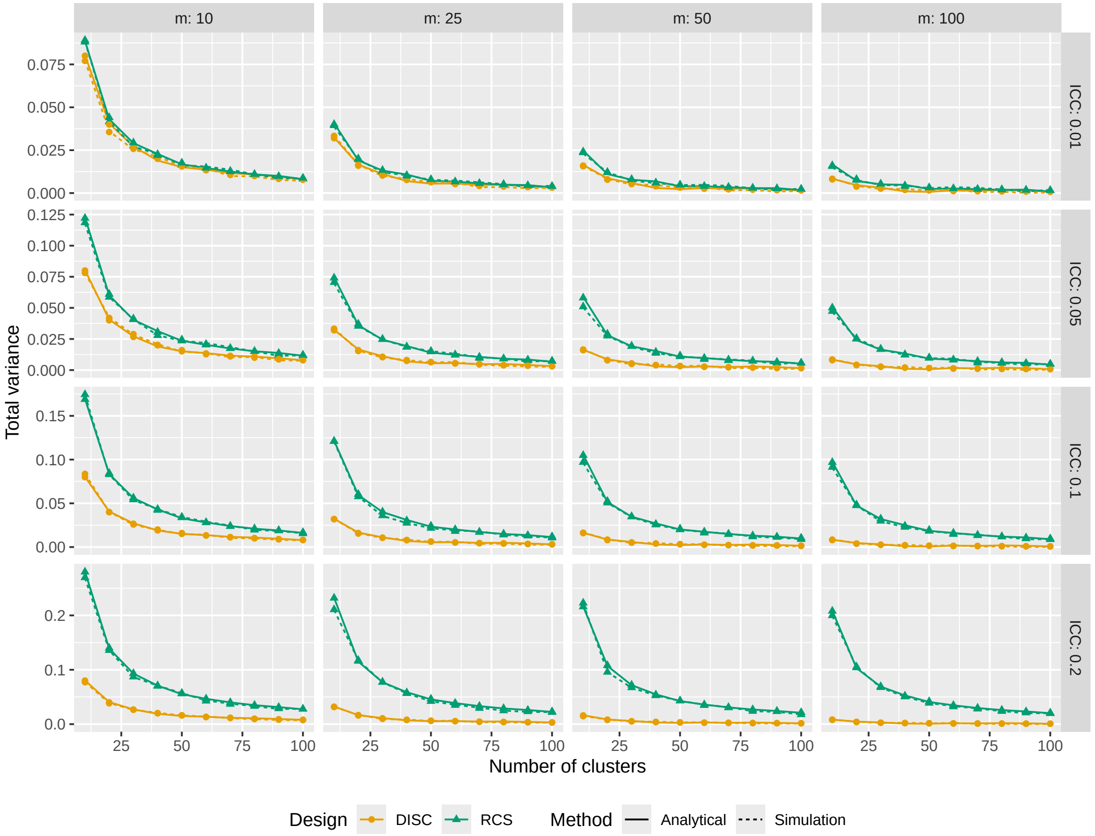


Figure S1: Estimated total variance of the linear treatment effect estimator as a function of the number of clusters under DISC (Different Individuals, Same Clusters) and RCS (repeated cross-sectional) designs. Dotted lines represent simulation-based variance and solid lines represent theoretical analytic variance. Green lines with triangle points represent RCS designs and orange lines with circular points represent DISC designs. The column facets correspond to four different cluster sizes (10, 25, 50, and 100) and the row facets correspond to four different ICC (intraclass correlation coefficient) values (0.01, 0.05, 0.1, 0.2).

Figure S2 shows how the variance of the linear estimator changes as we increase variation in cluster sizes. The same simulation setup is used, except cluster sizes were generated by sampling from a Normal distribution with mean 25 and a standard deviation of either 0 (implying no variation in cluster sizes), 4, or 8. Results are shown for two ICC values (0.01 and 0.1). These results show that the benefits of the DISC design relative to the RCS design persist even when cluster sizes are highly variable.

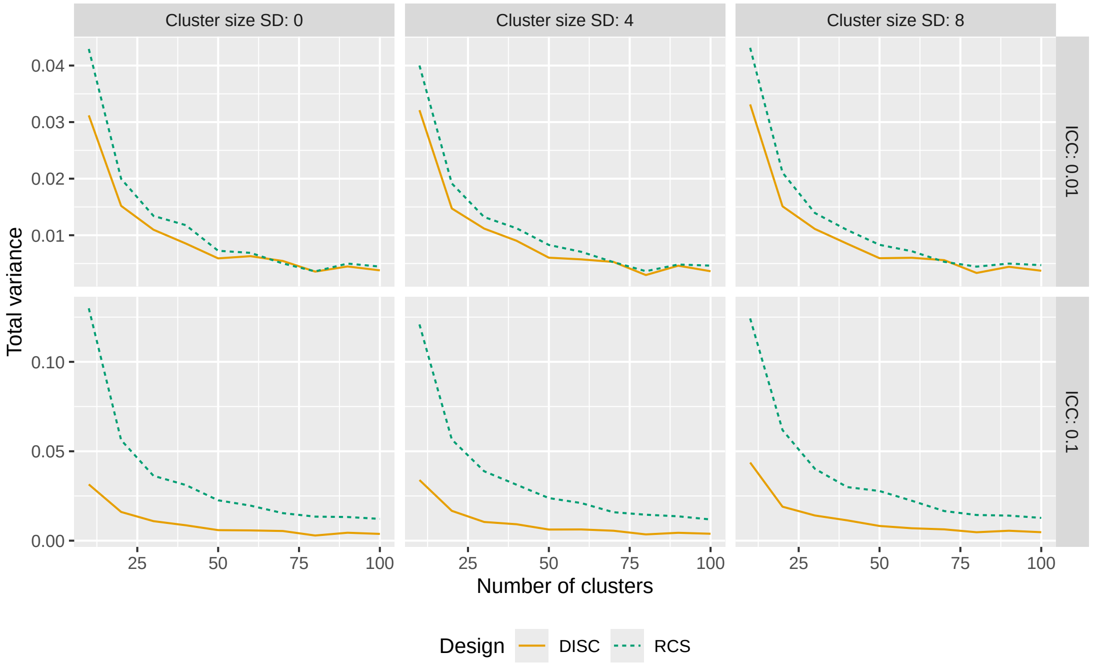


Figure S2: Total variance of the linear treatment effect estimator under DISC (Different Individuals, Same Clusters) and RCS (repeated cross-sectional) designs, estimated via simulation, as a function of the number of clusters. Dotted green lines represent RCS designs and solid orange lines represent DISC designs. The column facets correspond to three different cluster size standard deviations (0, 4, and 8) and the row facets correspond to two different ICC (intraclass correlation coefficient) values (0.01, and 0.1).

Figure S3 shows the total variance of both the linear and DRDID estimators under a three-level sampling design. Data were generated according to a three-level hierarchical model, with random intercepts at the first and second levels. Other than the additional random intercept at the second level and the sampling mechanism, the data-generating mechanism is identical to the one used for Figure 4. As expected, this figure shows that both versions of the DISC design (S-D-D and S-S-D) are superior to the RCS design (D-D-D) in terms of estimated variance. Furthermore, and also as expected, we see that variance is lower for the S-S-D design relative to the S-D-D design.

Finally, we give additional simulation results in Table S1, including estimated bias, variance, mean squared error, and 95% confidence interval coverage. These correspond to the simulation results displayed in Figure 3, with modifications in terms of the ICC, number of clusters, and the cluster sizes. The main purpose of displaying these results is to show that theoretical expectations match simulation results; we can see that the linear estimator is unbiased across all scenarios, that variance is roughly equal to MSE, and that nominal 95% confidence interval coverage is maintained.

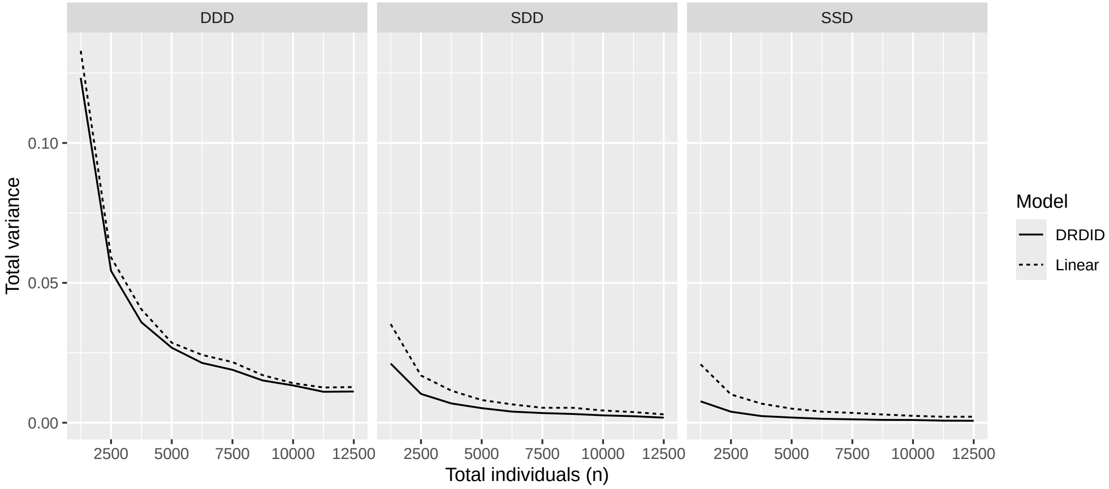


Figure S3: Estimated total variance under three different three-level sampling designs (Different-Different-Different, or D-D-D; Same-Different-Different, or S-D-D; and Same-Same-Different, or S-S-D), comparing a simple unadjusted linear model (dotted line) with a more complex adjusted doubly robust difference-in-differences model (solid line). Data are generated with a standard uniform covariate, 100 clusters, and an ICC (intraclass correlation coefficient) of 0.1.

Table S1: Simulation results for a subset of data-generating parameters across 1,000 simulation replicates, including bias, variance, mean squared error, and 95% confidence interval coverage.

| **Design** | **ICC** | **Number of clusters** | **Cluster size** | **Bias** | **Variance** | **MSE** | **Coverage** |
|---|---|---|---|---|---|---|---|
| DISC | 0.01 | 20 | 25 | 0.007 | 0.016 | 0.016 | 94.3% |
| DISC | 0.01 | 20 | 100 | 0.006 | 0.006 | 0.006 | 95.0% |
| DISC | 0.01 | 50 | 25 | 0.001 | 0.004 | 0.004 | 96.1% |
| DISC | 0.01 | 50 | 100 | 0.001 | 0.002 | 0.002 | 95.4% |
| DISC | 0.1 | 20 | 25 | 0.001 | 0.015 | 0.015 | 95.6% |
| DISC | 0.1 | 20 | 100 | -0.005 | 0.006 | 0.006 | 95.4% |
| DISC | 0.1 | 50 | 25 | 0.000 | 0.004 | 0.004 | 95.0% |
| DISC | 0.1 | 50 | 100 | -0.001 | 0.002 | 0.002 | 96.0% |
| RCS | 0.01 | 20 | 25 | -0.002 | 0.020 | 0.020 | 95.4% |
| RCS | 0.01 | 20 | 100 | -0.003 | 0.008 | 0.008 | 95.6% |
| RCS | 0.01 | 50 | 25 | -0.004 | 0.008 | 0.008 | 95.1% |
| RCS | 0.01 | 50 | 100 | -0.002 | 0.003 | 0.003 | 96.6% |
| RCS | 0.1 | 20 | 25 | -0.006 | 0.058 | 0.058 | 95.1% |
| RCS | 0.1 | 20 | 100 | 0.008 | 0.022 | 0.022 | 96.8% |
| RCS | 0.1 | 50 | 25 | 0.000 | 0.048 | 0.047 | 95.0% |
| RCS | 0.1 | 50 | 100 | -0.002 | 0.018 | 0.018 | 94.9% |
